# Comparison of Corneal Biomechanics in Post-SMILE, Post-LASEK, and Keratoconic Eyes

**DOI:** 10.3389/fmed.2021.695697

**Published:** 2021-09-14

**Authors:** Jianmin Shang, Yang Shen, Vishal Jhanji, Xueyi Zhou, Jing Zhao, Yu Zhao, Xingtao Zhou

**Affiliations:** ^1^Eye Institute and Department of Ophthalmology, Eye and ENT Hospital, Fudan University, Shanghai, China; ^2^NHC Key Laboratory of Myopia, Fudan University, Shanghai, China; ^3^Key Laboratory of Myopia, Chinese Academy of Medical Sciences, Shanghai, China; ^4^Shanghai Research Center of Ophthalmology and Optometry, Shanghai, China; ^5^Department of Ophthalmology, University of Pittsburgh School of Medicine, Pittsburgh, PA, United States

**Keywords:** keratoconus, SP-A1, LASEK, corneal biomechanics, small incision lenticule extraction

## Abstract

**Significance:** Our study found that SP-A1 (stiffness parameter at time of first applanation) was statistically different between post-laser-assisted subepithelial keratomileusis (LASEK) and post-small incision lenticule extraction (SMILE) eyes. The stiffness of keratoconus was lower than that of post-refractive surgery corneas.

**Purpose:** To compare corneal biomechanics among post-SMILE, post-LASEK, and keratoconic eyes.

**Methods:** In this retrospective study, 36 eyes of 36 patients after SMILE, 36 eyes of 36 patients after LASEK, and 36 eyes of 36 patients with keratoconus with matching (±5 μm) central corneal thickness (CCT) were examined using Scheimpflug corneal topography (Pentacam HR) and dynamic Scheimpflug analyzer (CorVis ST). Mixed linear model analysis with Bonferroni-adjusted *post-hoc* comparisons was performed to compare the differences in corneal biomechanics and topographic parameters among the three groups with the CCT and the bIOP (intraocular pressure with biomechanical correction) as the random factor.

**Results:** All groups had comparable CCT at baseline. The bIOPs between the three groups were comparable. The mean values of the Belin/Ambrósio Deviation (BAD-D) in the keratoconus group were significantly higher than those of the SMILE (*post hoc p* < 0.001) and LASEK groups (*post hoc p* < 0.001). The SP-A1 in the keratoconus group was the lowest when compared with those of the SMILE (*post hoc p* = 0.003) and LASEK groups (*post hoc p* < 0.001). The SMILE group SP-A1 values were slightly lower than those of the LASEK group (*post hoc p* = 0.044).

**Conclusions:** Keratoconus eyes were significantly softer when compared to post-refractive surgery corneas with comparable corneal thickness in terms of SP-A1 and BAD-D values, while the LASEK group may have the greatest stiffness. Post-SMILE and post-LASEK corneas showed significant differences in SP-A1.

## Introduction

Keratoconus is a bilateral, progressive ectatic disorder, which causes thinning of the corneal stroma, irregular astigmatism, and loss of best spectacle-corrected vision (1–3). The prevalence is around 1/2,000 in the general population (2). Another form of corneal ectasia is seen after refractive surgeries, mainly after laser *in situ* keratomileusis (LASIK) (4–6). On the other hand, small incision lenticule extraction (SMILE) (7, 8) and laser-assisted subepithelial keratomileusis (LASEK) are stromal flap-free surgeries, which are rarely associated with corneal ectasia. This might be attributed to the lack of corneal flap and other factors such as corneal stromal bed thickness (9). Post-SMILE and post-LASEK eyes are therefore good models for understanding the effect of tissue removal on the cornea.

It is generally believed that the compromise of corneal biomechanics precedes the occurrence of topographic abnormality (10). CorVis ST is a dynamic Scheimpflug analyzer, which is able to capture the corneal deformation process caused by an air puff. By analyzing the parameters including deformation amplitude, time, length, and velocity, *in-vivo* corneal biomechanics are assessed and quantified (9). The new Vinciguerra Screening Report provides parameters such as deformation amplitude ratio at 2 mm, (DAR-2mm), integrated inverse concave radius (IR), Ambrosio relational thickness horizontal (ARTh), and stiffness parameter at the first applanation (SP-A1) to describe corneal biomechanics. The present study intended to detect the difference in corneal biomechanics between the corneas that underwent flapless corneal refractive surgeries (SMILE and LASEK) keratoconus under the condition of the similar corneal thickness (and bIOP). A post-LASEK cornea (myopia correction) lacks Bowman's layer, and the central corneal thickness is much thinner than the peripheral. A post-SMILE cornea remains a relative intact anterior stroma, but a potential gap formed in the anterior stroma after the stromal lenticular extraction. In the present study, we compared the DAR-2mm, IR, ARTh, SP-A1, and Belin/Ambrósio Deviation (BAD-D) values (Table 1) among keratoconic, post-SMILE, and post-LASEK patients. We hope that through this study, we can better understand the corneal biomechanical differences between keratoconus and after corneal refractive surgery.

**Table 1:** Definitions of the abbreviations.

**Abbreviation**	**Definition**
DAR-2mm: deformation amplitude ratio 2mm	The ratio of deformation amplitude to apex at 2mm
IR: Intergrated inverse concave radius	The area under the curve of the inverse concave radius
ARTh: Ambrosio relational thickness horizontal	The thinnest pachymetry/pachymetric progression
SP-A1: stiffness parameter at time of first applanation)	The resulting pressure on the cornea at the time of applanation/deflection amplitude at this time (resulting load/deformation)
BAD-D: Belin/Ambrósio Deviation	BAD-D is the total parameter for keratoconus early detection. Values of over 1.6 indicate abnormal. Values of over 3.0 indicate extreme abnormal

## Patients and Methods

### Patients

In this retrospective study, we included participants who had undergone SMILE or LASEK procedure with matching (±5 μm) central corneal thickness (CCT). We also included patients with keratoconus. Keratoconus was diagnosed following the Global Consensus guidelines (11). Besides, the cones were within 4 mm in the center. One eye of each participant was selected. A total of 36 eyes of 36 patients with keratoconus (keratoconus group), 36 eyes of 36 participants who had undergone SMILE (SMILE group), and 36 eyes of 36 participants who had undergone LASEK (LASEK group) were enrolled from the Department of Ophthalmology, Eye and ENT Hospital of Fudan University. Among them, the keratoconus group was randomly selected from patients who visited our hospital in the recent 2 years and did not receive any eye surgical treatment, and the examination was measured immediately at the time of first visit as routine. As for the other two groups, we selected the patients who underwent refractive surgery who met the requirement of CCT, then selected them randomly, and then the examination was performed 2 years after the operation. In addition, some keratoconus patients failed to enter the study because of the difference of CCT between keratoconus patients and patients who underwent corneal surgery which was beyond 5 μm. The study protocol followed the tenets of the Declaration of Helsinki and was approved by the ethics committee of the Eye and ENT Hospital of Fudan University. An informed consent was obtained from all participants. The ethical committee approval code is KJ2010-18.

### Surgeries

All the procedures were performed by one surgeon (XTZ). All the target diopters were set to 0D. SMILE procedures were performed using a VisuMax femtosecond laser system (Carl Zeiss Meditec, Jena, Germany) with a pulse energy of 130 nJ. The cap was set to 120-μm thickness and 7.5-mm diameter. The side cut was set to 2 mm. The superior surface and the inferior surface of the lenticule were separated from the anterior stroma. The lenticule was then extracted through the side cut. LASEK was performed using a Mel 80 excimer laser (Carl Zeiss Meditec, Jena, Germany) system. Corneal epithelial trephines (Model 52503B; 66 Vision Tech Co., Ltd., Suzhou, China) with an inner diameter of 8.5 mm and a 20% ethanol-aqueous solution were employed to create an epithelial flap. The excimer laser with a repetition rate of 250 kHz and pulse energy of 150 nJ was used to ablate the corneal stroma. The epithelial flap was repositioned after the excimer laser treatment (9).

### Measurement

Each patient underwent manifest refraction spherical equivalent (MRSE), best spectacle-corrected distant visual acuity (BSCDVA), and slit-lamp examination. Measurements were performed preoperatively and 2 years postoperatively. Corneal topographies were captured using Pentacam HR. CCT from Pentacam was used for statistical comparison. Corneal biomechanical assessments were measured with CorVis ST. Pentacam and CorVis measurements were performed three times for each eye, and the quality of the image was OK. The DAR-2mm, IR, SP-A1, Belin/Ambrosio Deviation (BAD-D) values were exported (4, 5, 12–16).

### Data Analysis

Data analysis was performed using SPSS Version 22.0 software. The Kolmogorov–Smirnov test was used for normality tests. The Friedman test was used to analyze the differences in age, gender, CCT, MRSE, bIOP (intraocular pressure with biomechanical correction), Flat K, Steep K, and Kmax among the three groups. Mixed linear models with Bonferroni-adjusted *post-hoc* comparisons were used to analyze the differences in the mean values of DAR-2mm, IR, ARTh, SP-A1, and BAD-D with the operation mode as the fixed factor and the CCT and the bIOP as the random factors.

## Results

Table 2 summarizes the demographic characteristics in all groups. The mean age of patients in the SMILE, LASEK, and keratoconus groups was 31 ± 7 years, 28 ± 7 years, and 26 ± 6 years, respectively. The mean CCT values were 465.61 ± 29.22 μm, 463.22 ± 30.44 μm, and 464.83 ± 30.10 μm, respectively. The age of the patients (χ^2^ = 3.909, *p* = 0.141), gender (χ^2^ = 0.615, *p* = 0.735), left/right eyes (χ^2^ = 1.200, *p* = 0.549), CCT(χ^2^ = 0.093, *p* = 0.942), bIOP (χ^2^ = 4.325, *p* = 0.115), and MRSE (χ^2^ = 0.515, p = 0.767) were statistically comparable in all three groups. Flat K (χ^2^ = 21.248, *p* < 0.001) of the three groups were 38.63 ± 2.77, 38.82 ± 1.93, and 48.03 ± 6.31. Steep k (χ^2^ = 22.768, *p* < 0.001) were 39.31 ± 2.67, 39.87 ± 1.79, and 51.44 ± 6.70. Kmax (χ^2^ = 24.322, *p* < 0.001) were 43.88 ± 1.19, 43.45 ± 2.58, and 59.11 ± 11.14, respectively.

**Table 2:** Demographic characteristics.

			**SMILE**	**LASEK**	**KC**	**χ2**	**P**
Age (years)	Mean ± SD		31 ± 7	28 ± 7	26 ± 6	3.909	0.141
Gender	Male	Count	14	18	14		
		%	38.9%	50.0%	38.9%		
	Female	Count	22	18	22	0.615	0.735
		%	61.1%	50.0%	61.1%		
Eye	Right	Count	20	26	26		
		%	55.6%	72.2%	72.2%		
	Left	Count	16	10	10	1.200	0.549
		%	44.4%	27.8%	27.8%		
CCT (μm)	Mean ± SD		465.61 ± 29.22	463.22 ± 30.44	464.83 ± 30.10	0.093	0.942
bIOP (mmHg)	Mean ± SD		14.57 ± 1.33	14.52 ± 1.56	15.88 ± 2.62	4.325	0.115
	95% CI		13.56–15.66	13.06–15.07	14.37–18.54	-	-
Flat K	Mean ± SD		38.63 ± 2.77	38.82 ± 1.93	48.03 ± 6.31	21.248	<0.001^**^
	95% CI		36.78–40.66	33.69–42.31	43.55–52.81	-	-
Steep K	Mean ± SD		39.31 ± 2.67	39.87 ± 1.79	51.44 ± 6.70	22.768	<0.001^**^
	95% CI		37.46–41.23	35.34–42.12	47.36–56.47	-	-
Kmax	Mean ± SD		43.88 ± 1.19	43.45 ± 2.58	59.11 ± 11.14	24.322	0.000^**^
	95% CI		43.04–44.01	35.18–47.76	51.08–69.25	-	-
MRSE (D)	Mean ± SD		−7.19 ± 4.88	−7.25 ± 1.09	−7.99 ± 1.90	0.515	0.767
	95% CI		−10.47– −3.92	−9.96– −4.54	−11.75– −4.22	-	-

*CCT, central corneal thickness; MRSE, manifest refraction spherical equivalent; KC, Keratoconus; μm, micron; D, diopter*.

** *P < 0.01*.

**Figure 1:**
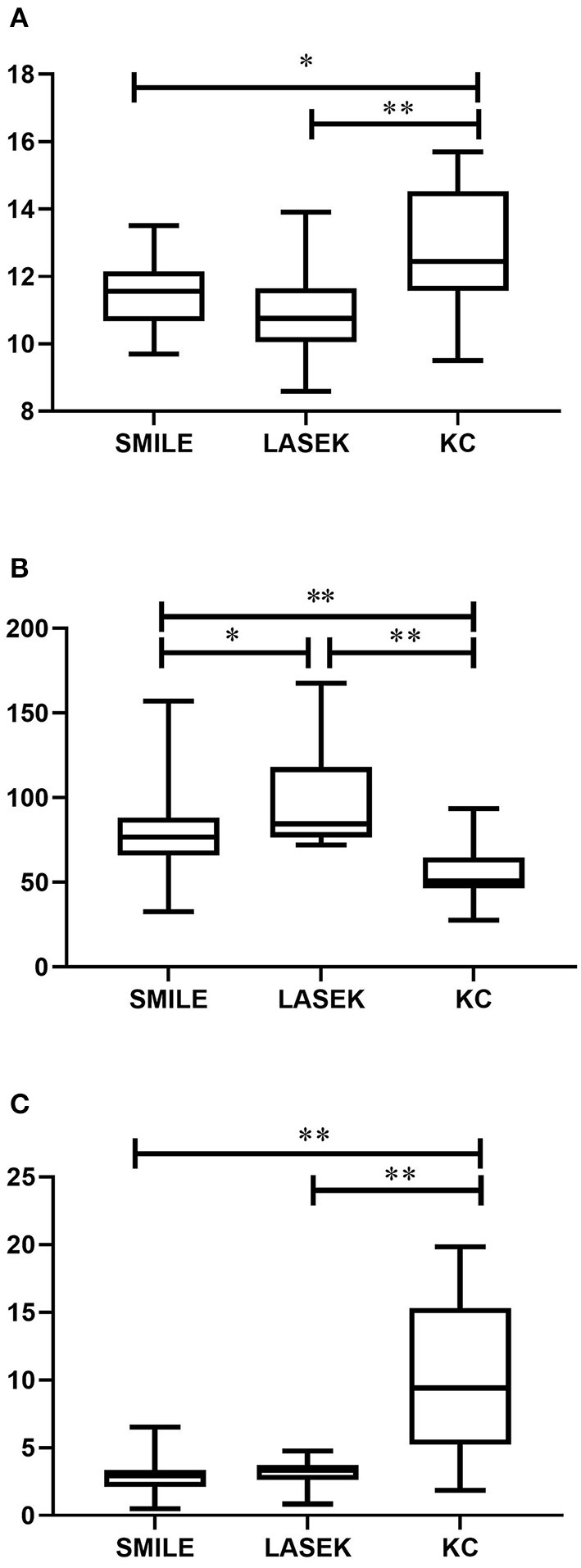
Corneal biomechanics among post-SMILE, post-LASEK, and keratoconic eyes. **(A)** IR; **(B)** SP-A1; **(C)** BAD-D. SMILE, small incision lenticule extraction; LASEK, laser-assisted subepithelial keratomileusis; KC, keratoconus. ^*^P < 0.05 and ^**^P < 0.01.

The biomechanical parameters are listed in Table 3. Overall, statistically significant differences were detected in the mean values of SP-A1 between post-refractive surgery and keratoconus eyes (*p* < 0.05). Figure 1A shows that the mean IR values in the LASEK group and in the SMILE group were significantly lower than those in the keratoconus group (*post hoc* P (LASEK vs. keratoconus) < 0.001, *post hoc* P (SMILE vs. keratoconus) = 0.014).

**Table 3:** Main corvis ST parameters.

**Parameters**	**Groups**						***F*-value**	***P*-value**	***Post-hoc* P**	***Post-hoc* P**	***Post-hoc* P)**
									**(SMILE vs. KC)**	**(LASEK vs. KC)**	**(SMILE vs. LASEK)**
	**SMILE**		**LASEK**		**KC**						
	**Mean ± SD**	**95% CI**	**Mean ± SD**	**95% CI**	**Mean ± SD**	**95% CI**					
DAR-2mm	5.59 ± 0.43	5.18–5.93	5.17 ± 0.55	4.85–5.62	5.88 ± 1.13	5.47–6.20	2.572	0.093	0.923	0.059	0.301
IR(ms · mm)	11.48 ± 1.17	10.88–12.31	10.88 ± 1.28	10.24–11.71	12.70 ± 1.95	12.09–13.48	10.336	<0.001^**^	0.014^*^	<0.001^**^	0.338
ARTh(μm)	181.57 ± 62.34	144.60–207.07	192.39 ± 69.95	166.92–230.48	193.44 ± 66.80	169.06–230.79	1.478	0.495	0.877	1.000	0.676
SP-A1(mmHg/mm)	78.42 ± 27.55	65.51–89.45	98.32 ± 28.76	88.28–112.92	55.02 ± 15.98	42.51–65.77	16.714	<0.001^**^	0.002^**^	<0.001^**^	0.044^*^
BAD-D	3.01 ± 1.71	1.45–4.75	3.08 ± 1.14	1.38–4.77	10.22 ± 5.44	8.55–11.75	43.517	<0.001^**^	<0.001^**^	<0.001^**^	1.000

*KC, Keratoconus; DAR-2mm, ratio of deformation amplitude to apex at 2 mm; IR, intergrated inverse concave radius; ARTh, Ambrósio Relational Thickness to the horizontal profile; SP-A1, stiffness parameter at the first applanation; BAD-D, Belin/Ambrósio Deviation; ms, millisecond; mm, millimeter;*

* *P < 0.05*.

** *P < 0.01*.

Statistically significant differences were also noted between LASEK and SMILE groups in SP-A1 (*post hoc p* = 0.044) but not in IR (*post hoc p* = 0.338) (Figure 1B). Figure 1C shows the mean BAD-D values in all groups. The keratoconus group had significantly higher BAD-D values when compared to SMILE (*post hoc p* < 0.001) and LASEK groups (*post hoc p* < 0.001). The difference in the mean BAD-D values between the SMILE and LASEK groups was not significant statistically (*post hoc p* = 1.000).

The differences in the mean values of the DAR-2mm and ARTh were not significant among the three groups (*F* = 2.572; *p* = 0.093 and *F* = 1.478, *p* = 0.495, respectively).

## Discussion

It is hypothesized that alterations in corneal biomechanics and bIOP play a key role in primary and post-LASIK corneal ectasia (17). Therefore, measurement of biomechanical parameters is important for identification of corneal ectasia at an early stage (18, 19). The present study investigated the differences in corneal topography and biomechanics in keratoconus and post-refractive surgery eyes using CorVis ST and Pentacam HR platforms. We noted that SP-A1 was the lowest in the keratoconus group, while it was highest in the LASEK group. SP-A1 describes the deformation resistance of the cornea (4). Our findings indicated that for matched CCT, bIOP, and age, corneas of keratoconus patients were less “stiff” than those of patients who underwent LASEK or SMILE. Simultaneously, the mean IR value was the lowest in the LASEK group and highest in the keratoconus group, again indicating the degree of compromised biomechanics in keratoconus patients. These findings are consistent with previous reports showing that keratoconus is characterized by decreasing corneal thickness, disruption of Bowman's layer, and disintegration of corneal collagen lamellae (20). The difference of SP-A1 between the SMILE group and LASEK group may be related to the stromal layer removed during SMILE operation, while LASEK retained the integrity of the stromal layer. When detecting SP-A1, its essence is to measure the deformation degree of the cornea when it is stressed and sunken by air impact. For LASEK, the cornea will deform as a whole when it is impacted, but SMILE is not. Due to the existence of a cap, a potential gap will be formed between the cap and the residual corneal stroma. Although the selected patients after SMILE have been followed up for 2 years, theoretically, this gap cannot heal, and the existence of this gap may be the reason for the greater deformation of the cornea when impacted by force.

We observed in our study that DAR-2mm and ARTh were similar among the three groups. DAR-2mm is the ratio of deformation amplitude at the apex of the cornea to a point 2 mm away from the apex. ARTh is the quotient of corneal thickness at the thinnest point of the horizontal meridian and the thickness progression (16). For keratoconus, the CCT is much lower than the peripheral corneal thickness. The deformation amplitude in the central zone is larger than the peripheral zone, and hence the DAR-2mm value is higher in keratoconic eyes when compared to the normal eyes. Meanwhile, keratoconus has larger pachymetric progression than normal eyes. Since ARTh equals to the thinnest pachymetry divided by pachymetric progression, the value is lower in keratoconic eyes. The DAR-2mm and ARTh values could identify keratoconus from otherwise normal thin corneas. However, after corneal refractive surgery for myopia correction, more tissue would be ablated from the central zone of the cornea compared to the peripheral zone. As the central thickness would decrease more than the peripheral thickness, the deformation amplitude in the central zone would increase significantly, and thus the DAR-2mm value would increase. The ARTh value would also decrease postoperatively owing to a decrease in pachymetry in the central zone after myopia correction, which increases the pachymetric progression. The mean BAD-D value in the keratoconus group was much higher than those of the SMILE and LASEK groups, which corresponded with SP-A1 values among the three groups.

In our study, it was noteworthy that there was no significant difference in BAD-D values between SMILE and LASEK groups, but the LASEK group had higher SP-A1 values when compared with those of the SMILE group. Reinstein et al. established a mathematical model to investigate the effects of photorefractive keratectomy (PRK), LASIK, and SMILE on corneal biomechanics. It was reported that theoretically, SMILE should lead to a minimal effect on corneal biomechanics when compared with PRK and LASIK as the integrity of the anterior stroma is maximally maintained (21). However, in recent years, studies have shown that PRK had small effect on central biomechanics when compared with SMILE (22), which suggests that our biomechanical research needs to be further developed. In our study, we found that the SP-A1 value in the SMILE group was significantly lower than that in the LASEK group, indicating that overall the effect of LASEK on corneal biomechanics is less when compared to SMILE. SP-A1 describes the stiffness of cornea at the time of first applanation. In our previous study, it is possible that the potential gap between the cap and stromal bed after SMILE, which could be detected with Scheimpflug camera (23), may be a confounding factor that influences the accuracy of assessment in early stage. The patients selected in this study were assessed 2 years after surgery, and no obvious gap was found during the follow-up. Therefore, the cap/bed interface between two stressed regions after SMILE may be the biggest difference from LASEK. Further research is still necessary.

We originally considered that keratoconus stage may have an impact on corneal biomechanics, but in order to match CCT, which is different in patients after corneal refractive surgery, and CCT is also an important factor in keratoconus stage, we only matched CCT and bIOP which have great impact on corneal biomechanics.

The major limitation of our study is the small sample size. Studies with a larger sample size and long-term follow-up are recommended. Besides being a retrospective study and lacking preoperative parameters, the study may produce a degree of deviation, which needs further research in the future. Finally, in order to better match CCT, this study failed to unify the stage of keratoconus. This is also due to the limited sample size of this study. In the future, this point should be paid more attention.

In conclusion, our study found that SP-A1 was statistically different between post-LASEK and post-SMILE eyes. The stiffness of keratoconus was lower than that of post-refractive surgery corneas.

## Data Availability Statement

The raw data supporting the conclusions of this article will be made available by the authors, without undue reservation.

## Ethics Statement

The studies involving human participants were reviewed and approved by the ethics committee of the Eye and ENT Hospital of Fudan University. The patients/participants provided their written informed consent to participate in this study.

## Author Contributions

JS: experimental design, data collection, and writing manuscript. YS: experimental design and data collection. VJ: data analysis and article embellishment. XuZ: data collection and analysis. JZ and YZ: design of experimental ideas. XiZ: experimental ideas design guidance, funds supply, and equipment supply. All authors contributed to the article and approved the submitted version.

## Funding

This work was supported in part by the National Natural Science Foundation of China (Grant No. 81770955), the Joint Research Project of New Frontier Technology in Municipal Hospitals (SHDC12018103), the Project of Shanghai Science and Technology (Grant No. 20410710100), the Major Clinical Research Project of Shanghai Shenkang Hospital Development Center (SHDC2020CR1043B), and the Project of Shanghai Xuhui District Science and Technology (2020-015). The funders had no role in study design, data collection and analysis, decision to publish, and preparation of the manuscript.

## Conflict of Interest

The authors declare that the research was conducted in the absence of any commercial or financial relationships that could be construed as a potential conflict of interest. The handling editor is a consultant for the manufacturer Corvis ST.

## Publisher's Note

All claims expressed in this article are solely those of the authors and do not necessarily represent those of their affiliated organizations, or those of the publisher, the editors and the reviewers. Any product that may be evaluated in this article, or claim that may be made by its manufacturer, is not guaranteed or endorsed by the publisher.
